# Recent Advances in Solid-Electrolyte Interphase for Li Metal Anode

**DOI:** 10.3389/fchem.2022.916132

**Published:** 2022-05-20

**Authors:** Dafang He, Junhong Lu, Guangyu He, Haiqun Chen

**Affiliations:** Key Laboratory of Advanced Catalytic Materials and Technology, Advanced Catalysis and Green Manufacturing Collaborative Innovation Center, Changzhou University, Changzhou, China

**Keywords:** lithium-ion battery, Li metal anode, lithium dendrite, SEI layer, modification

## Abstract

Lithium metal batteries (LMBs) are considered to be a substitute for lithium-ion batteries (LIBs) and the next-generation battery with high energy density. However, the commercialization of LMBs is seriously impeded by the uncontrollable growth of dangerous lithium dendrites during long-term cycling. The generation and growth of lithium dendrites are mainly derived from the unstable solid–electrolyte interphase (SEI) layer on the metallic lithium anode. The SEI layer is a key by-product formed on the surface of the lithium metal anode during the electrochemical reactions and has been the barrier to development in this area. An ideal SEI layer should possess electrical insulating, superior mechanical modulus, high electrochemical stability, and excellent Li-ion conductivity, which could improve the structural stability of the electrode upon a long cycling time. This mini-review carefully summarizes the recent developments in the SEI layer for LMBs, and the relationship between SEI layer optimization and electrochemical property is discussed. In addition, further development direction of a stable SEI layer is proposed.

## Introduction

Lithium-ion batteries (LIBs) are the dominant power source for electric vehicles and large-scale renewable energy storage systems (He et al., 2022). However, the energy density of widely used LIBs has reached 300 WhKg^−1^ and is closed to its theoretical values, which is far from the demand for ever-growing energy density (Meyerson et al., 2021). Therefore, it is urgent to explore a new materials system with high specific capacity. Lithium metal has attracted much attention owing to its high specific capacity (3,860 mAhg^−1^), low mass density (0.59 g cm^−3^), and the lowest electrochemical reaction potential (−3.040 V vs. standard hydrogen electrode) (Haiping Wu et al., 2021). A high energy density of 600 Whkg^−1^ can be achieved when lithium metal anode is matched with intercalation-type cathode materials (such as NCM and NCA) (Shan et al., 2021). The theoretical energy density can be further enhanced to 2,600 and 3500 Whkg^−1^ when the Li metal anode is used in Li-S and Li-O_2_ battery systems (Liping Wang et al., 2019). Therefore, lithium metal batteries (LMBs) are considered to be a substitute for LIBs and the next-generation battery with high energy density.

The commercialization of LMBs is seriously impeded by poor cycling stability and serious safety issues, which are caused by the uncontrollable growth of dangerous lithium dendrites during continuous cycling. During the first charge process, a solid–electrolyte interphase (SEI) layer forms when active lithium metal reacts spontaneously with organic electrolyte (Jiang et al., 2020). The main role of SEI layer is to avoid direct exposure of the Li metal to electrolyte and prevent the endless parasitic reactions, which could improve the stability of the Li metal anode in an organic solvent. However, the SEI layer undergoes repeated destruction/reconstruction due to the huge volume expansion of lithium metal during the charging process, leading to continuously consume electrolyte and lithium metal anode by the formation of a new SEI layer, which results in a rapid capacity fade upon cycling (Wei et al., 2018). The most serious is that the unstable SEI layer could cause nonuniform Li deposition, thus promoting the growth of lithium dendrites. The continuous growth of lithium dendrites may cause a short circuit, which is the biggest potential safety hazard in lithium metal anode toward commercialization (Chen et al., 2021).

To overcome these obstacles, a modified SEI layer is one of the most promising routes to inhibit the growth of lithium dendrite and alleviate drastic volumetric variation, thus achieving outstanding electrochemical properties. In this mini-review, the modification of SEI layer for metallic lithium electrode in recent years are summarized and discussed. First, the formation process and mechanism of SEI layers are discussed in brief. Second, the recent advances in constructing inorganic SEI layers, organic SEI layers, and inorganic/organic hybrid SEI layers are systematically reviewed. Finally, the remaining challenges and outlooks of modified SEI layers are evaluated.

## Formation Process and Mechanism of SEI Layer

The SEI layer, first proposed by Peled in 1979, refers to a passivation layer formed on the Li metal surface during cycling (Peled, 1979). The SEI layer was spontaneously formed *in situ* on the Li metal anode when the high chemical reactive Li metal was exposed to organic electrolytes. The widely accepted SEI layer structure is presented by the mosaic model, which is composed of inorganic components (Li_2_O, LiOH, LiF, Li_2_CO_3,_ and hydrocarbons) inside and organic components outside (Peled et al., 1997). According to the mosaic model, the SEI layer is complex in composition, which easily causes nonuniform Li deposition. An intrinsic SEI layer is electron insulating and Li-ion conductive, which could prevent the continuous reaction between metallic lithium electrodes and electrolytes (Hou et al., 2020). The desired SEI should possess electron insulation, high lithium ions diffusivity, superior mechanical modulus, favorable elasticity, and homogeneous composition to inhibit the growth of treelike lithium dendrites and maintain the controllable interface stability upon repeated cycling (Zhenxing Wang et al., 2021). Many advanced characterization methods (such as cryo-electron microscopy and secondary ion mass spectrometry) have been developed to study the formation process and microstructure evolution of SEI layer during repeated cycling (Ju et al., 2021; Yujing Liu et al., 2021). However, the *in situ* defective SEI layer usually suffers from inferior Li-ions diffusivity, low mechanical strength, and nonuniform composition, which make it difficult to accomplish flat and stable electroplating/stripping of metallic lithium anode (Aslam et al., 2021). The uneven SEI layer will cause a nonuniform Li-ions flux and result in the subsequently inhomogeneous Li deposition, which promotes the generation of lithium dendrites. Lithium dendrites could puncture the fragile SEI layer with inferior mechanical strength, and a fresh SEI layer is then formed stemming from the reaction at the electrolyte−exposed metallic lithium anode interface. During the de-lithiation process, lithium dendrites are likely to segregate from the lithium metal anode surface and become “dead lithium,” leading to a relatively low Coulombic efficiency (CE). In addition, the continuous and uncontrollable growth of dangerous lithium dendrites may penetrate the membranes after long cycles, causing short-circuits and serious safety issues (Ding et al., 2021).

## Strategies to Modify Li Metal Anode

**SCHEME 1:**
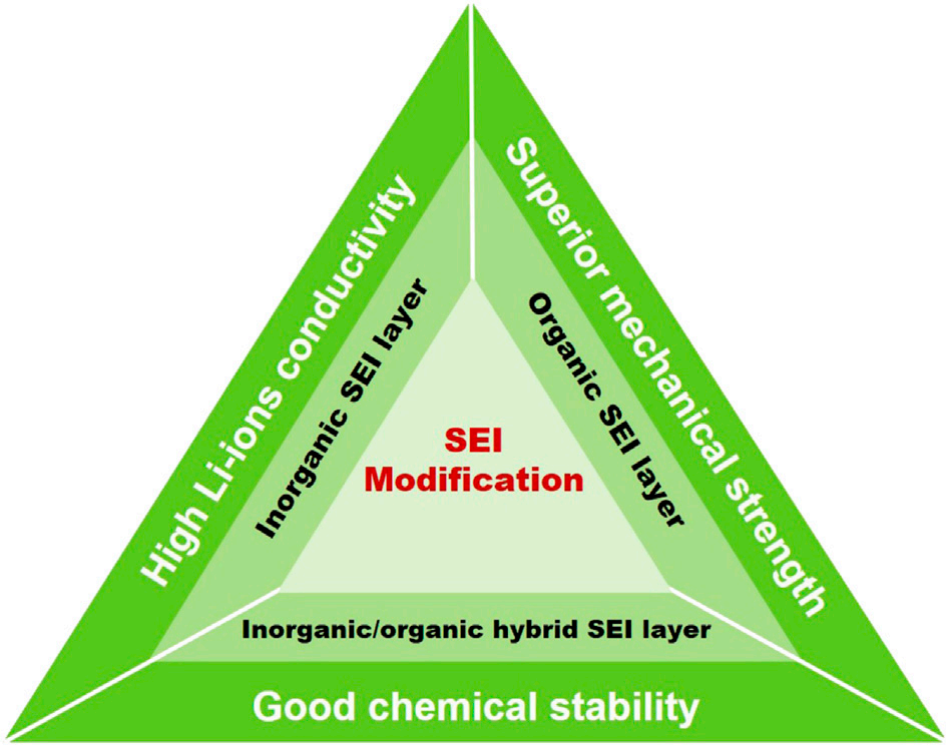
Schematic diagram of the solid–electrolyte interphase (SEI) modification strategy.

Based on the aforementioned discussions, it is found that the SEI layer is crucial for a stable lithium metal anode with long life cycling performance. High ionic conductivity, electrical insulating, and good mechanical toughness are indispensable characteristics for a desired SEI layer, which is beneficial for homogeneous Li deposition, imparting a rapid diffusion pathway for Li^+^ and restraining the generation of lithium dendrites (Hansen Wang et al., 2019; Qian et al., 2020). Therefore, designing and constructing a modified SEI layer is an effective approach to address the drawbacks encountered with metallic lithium anode. Up to date, kinds of inorganic, organic, and inorganic/organic hybrid SEI layers have been designed and constructed. In this section, we aim to carefully discuss the recent advances realized by the modification of SEI layers (Scheme 1, Table 1).

**TABLE 1:** Properties of the inorganic, organic, and inorganic/organic hybrid SEI layers.

SEI layer	Advantages	Disadvantages
Inorganic layer	High ionic conductivity	Low mechanical properties
Organic layer	High mechanical properties	Inferior ionic conductivity
Inorganic/organic hybrid SEI layer	High ionic conductivity	—
	Superior mechanical properties	

### Inorganic SEI Layers

The continuous growth of lithium dendrites could be inhibited by a SEI layer with superior mechanical strength. Therefore, inorganic SEI layers could inhibit the lithium dendrites growth because of their high electrochemical stability (Chen et al., 2022). High Li-ion conductivity is another significant characteristic of an outstanding modified SEI layer as it can help suppress the generation of lithium dendrites *via* homogenizing Li-ion flux. Ceramics, metal sulfides, Li_3_N, halide salts, and two-dimensional (2D) materials are the typical interfacial coatings of inorganic SEI layers (Sufu Liu et al., 2021; Tan et al., 2021).

Ceramics generally have high mechanical strength, which can protect lithium metal anode and improve interfacial compatibility (Han et al., 2017; Kim et al., 2018). Alaboina et al. prepared a ZrO_2_ passivation film on Li metal anode by an atomic layer deposition (ALD). The as-obtained ZrO_2_ film is compact, smooth, and uniform, which facilitates Li-ion transport and causes homogenous distribution of Li^+^ to inhibit lithium dendrites growth. Therefore, ZrO_2_ modified lithium metal anode exhibits excellent thermal tolerance up to ∼180°C and rate performance (Alaboina et al., 2018). Recently, an ultra-thin titanium dioxide (TiO_2_) coating with a thickness of 20 nm was deposited on Cu foil *via* an ALD method. The lithiophilic feature of TiO_2_ layer lowers the Li nucleation overpotential, ensuring homogenous Li nucleation and deposition. As a result, an improved CE of 97.4% over 100 cycles at 1 mA cm^−2^ can be realized (Tan et al., 2020).

Metal sulfides generally possess high Li-ion conductivity, which can not only stabilize the lithium metal anode but also promote the Li-ion migration (Cha et al., 2018). Liu et al. prepared a mixed conductive Li_2_Se/Li_2_S layer on the surface of a lithium metal anode by gas-solid reaction between Li and SeS_2_. Compared with Li_2_S, Li_2_Se has better ionic conductivity, which facilitates Li-ion diffusion, regulates its uniform deposition, and effectively inhibits the growth of lithium dendrites. As a result, the Li_2_S/Li_2_Se-protected metallic lithium anode displays an excellent electrochemical performance over 900 h at a high current density of 3 mA cm^−2^ (Liu et al., 2020).

The homogeneous defect-free Li_3_N film has not only high ionic conductivity but also good strength, which can effectively inhibit the growth of dendrites (Guo et al., 2019; Zhong et al., 2020; Lu et al., 2021). Wang et al. constructed an intermolecular Li-N bond reinforced [LiNBH]_*n*_ SEI layer *via* dehydrogenation polymerization. Benefiting from its polymer-like structure, the [LiNBH]_*n*_ SEI layer is mechanically elastic and effectively alleviates the volume change during cycling. In addition, the [LiNBH]_*n*_ layer has good ionic conductivity and electronic insulation originating from the polar Li-N bonds, which is beneficial in regulating the deposition of Li-ion on the surface. Consequently, the [LiNBH]_*n*_ protected lithium metal anode greatly inhibits dendrite formation and shows good cycle stability of 700 h at 3 mA cm^−2^ (Wang et al., 2020).

A large number of studies show that the uniform and tough halides film can effectively improve the chemical stability of lithium metal, alleviate volume change, and inhibit the formation of lithium dendrites during repeated cycling (Xu et al., 2020; Zhang et al., 2021; Hagopian et al., 2022). Yan et al. fabricated a Cu/LiF mixed conductor interface phase (MCI) film on the Li metal surface through a controllable displacement reaction between lithium metal anode and CuF_2_ solution at room temperature. The as-obtained MCI film realizes the preferential deposition of Li-ion at the boundary regions between Cu and LiF, resulting in improved homogeneity and mechanical strength. Moreover, the MCI film enhances the ionic conductivity with Cu atoms acting as a destructor. These excellent features of MCI film effectively inhibit the formation of lithium dendrites, reduce the interface resistance, and prolong the cycle life (Yan et al., 2018).

Two-dimensional (2D) materials were generally used to fabricate three-dimensional (3D) hosts or Li^+^ redistributors for dendrite-free metallic lithium anode (Zhou et al., 2020a; Fan et al., 2021). Zhou et al. constructed a protective layer on the surface of *in situ* SEI layer using 2D honeycomb parallelly aligned MgO nanosheets. The uniform distributed pores on MgO nanosheets redistribute the Li-ion flux in the electrolyte. Meanwhile, a Li-Mg alloy layer was formed on the Li metal surface through Li-ion interaction, which facilitated the fast Li-ion diffusion and uniform distribution of Li-ion. Consequently, the modified lithium metal anode effectively suppressed the formation of lithium dendrites, leading to an improved CE of ∼99% and long-term cycle stability of 2,500 h at 10 mA cm^−2^ (Zhou et al., 2020b). Carbon fiber, graphene, and graphite materials can also be directly used to modify lithium metal (Ju et al., 2022). Bai et al. developed a spray-painting method to achieve a homogeneous reduced graphene oxide film on lithium metal using graphene oxide dispersion, which inhibits the growth of needle-like dendrites. The resultant lithium metal anode can run up to 1,000 cycles at 5 mA cm^−2^ without a short circuit (Bai et al., 2018).

### Organic SEI Layers

Compared with inorganic SEI layers, organic SEI layers are elastic and can alleviate the huge volumetric variation during repeated cycling, which effectively inhibit the growth of lithium dendrites and enhance the cycling stability of LMBs (Zheng et al., 2020; Jingyi Wu et al., 2021; Wang et al., 2022). Li et al. prepared a flexible Li polyacrylic acid (LiPAA) enhanced SEI layer by self-adapting interface regulation. With the help of the high binding ability and superior stability of LiPAA polymer, the modified SEI layer can effectively inhibit the parasitic reactions and address the safety issues. An excellent cycling performance of 700 h is realized in the modified lithium metal anode (Li et al., 2018). Chen et al. prepared a covalent organic framework (COF) film on the surface of lithium metal with a thickness of ∼10 nm, which effectively shortened the diffusion path of Li^+^ and improved the transfer efficiency. The unique microporous structures and large specific surface area of the COF film can redistribute the Li-ion flux and lead to the uniform deposition/stripping process. Meanwhile, the enhanced Young’s modulus of 6.8 GPa derived from ultrathin COF film can suppress the growth of lithium dendrites. As a consequence, the resulting anode exhibited improved cycling stability of 400 h at a high current density of 1 mA cm^−2^ (Chen et al., 2020).

Recently, a tough polyrotaxane-co-polyacrylic acid (PR-PAA) polymer with a slide-ring structure was constructed as a self-adaptive interfacial layer on a lithium metal anode. In this novel structure, a cross-linked network was formed *via* polyrotaxane (a-cyclodextrin) rings covalently bonded to PAA chains, which can move freely to maintain their toughness and fracture resistance, thus leading to a lower tension caused by Li dendrites growth. Benefiting from the slide-ring structure, PR-PAA is highly stretchable, flexible, and exhibits an ultrafast self-healing property which allows even cracked Li to remain intact without disintegrating upon continuous cycling. As a consequence, the modified lithium metal anode exhibits superior cycling stability for 1,000 h at a high current density of 6 mA cm^−2^ (Gao et al., 2021). Chang et al. prepared a modified SEI layer with planar polycyclic aromatic hydrocarbons (PAHs) coating by a simple synthetic approach. The as-obtained dihydroxyviolanthrone (DHV) film can effectively protect the SEI layer from electrolyte corrosion. Moreover, the oxygenic functional groups in the flexible and homogenous SEI layer can control the transport of Li^+^ to homogenize the Li deposition. The resultant SEI layer significantly enhances the CE and displays outstanding cycling stability of over 1,000 h at a high current density of 4 mA cm^−2^ (Shaozhong Chang et al., 2022).

### Inorganic/Organic Hybrid SEI Layers

Based on the aforementioned discussions, it is known that inorganic SEI layers generally possess good mechanical strength and high ionic conductivity, while organic SEI layers commonly have excellent elasticity. Single inorganic and organic SEI layers cannot satisfy the requirements of lithium metal anode. Therefore, an inorganic/organic hybrid SEI layer combined with the characteristics of organic and inorganic SEI layer is expected to achieve good ionic conductivity, mechanical strength, and elasticity (Jian Wang et al., 2021; Sun et al., 2021; Sun et al., 2022). Liu et al. sprayed the molten metallic lithium using the modified ether-based precursor solution. After cooling rapidly, a hybrid film composed of LIF, Li_3_N, and Li-containing organic substances was prepared on the surface of lithium metal anode. The organic substances ensured the integrity of the film and connected the inorganic particles with each other. The structure of the film is uniform and dense, and has good ion conductivity and chemical stability, which effectively slow down the side reaction and suppress the formation of lithium dendrites. As a consequence, the resulting anode exhibited an improved CE of 98.15% over 200 cycles and small hysteresis of <450 mV at a high current density of 10 mA cm^−2^ (Liu et al., 2019).

Polymers possessed better strength and elasticity compared with small organic molecules. Gao et al. constructed a polymer-inorganic SEI layer using reactive polymer complex (RPC) as a precursor, which was composed of poly(vinylsulfonyl fluoride-ran-2-vinyl-1,3-dioxolane) (P(SF-DOL)) and graphene oxide (GO) sheets. The obtained SEI layer consists of polymeric Li salts embedded with LiF nanoparticles and GO sheets. The *in situ* formed SEI layer is dense and chemical stable, which effectively inhibits the side reactions of lithium metal anode. The GO sheets enhance the mechanical strength and prevent the growth of lithium dendrites. The unreacted RPC can act as a buffer layer to protect the SEI layer. As a result, the efficiency of Li deposition can reach 99.1% at a current density of 4 mA cm^−2^ (Gao et al., 2019). Recently, a self-repairing and Li-ion conductive hybrid SEI layer was constructed using the cross-linked poly(dimethylsiloxane) and further optimized by adding SiO_2_ nanoparticles as reinforcement fillers. The introduction of SiO_2_ was helpful in significantly improving the Li-ion conductivity and the mechanical strength, which effectively inhibited the lithium dendrite growth and facilitated the Li depositing/stripping kinetics. The modified lithium metal anode exhibits improved cycling stability for 1,340 h at a current density of 0.5 mA cm^−2^ and 1.0 mA cm^−2^ (Caiyun Chang et al., 2022).

## Conclusion and Outlook

LMBs are considered to be a substitute for LIBs and the next-generation battery with high energy density. The continuous growth of lithium dendrites is the biggest potential safety hazard of lithium metal anodes toward commercialization. Although great progress has been achieved, there are still some bottlenecks in translating research results into practice application: 1) Even though the modified SEI layers significantly improves the cycling stability of lithium metal anodes, it still fails after long-term cycling and the CE is still insufficient for commercial applications; 2) much of the current research is limited in the laboratory. It is highly urgent to solve the problems of feasibility and cost before achieving the large-scale production; 3) the current characterization techniques for the SEI formation process and microstructure evolution are not comprehensive; and 4) the current evaluation of electrochemical performance is not comprehensive, such as thermal monitoring management and life prediction *via* simulating the actual service conditions.

Aiming at the gap between the current theoretical research and practical application, the research direction of lithium metal anodes in the future should focus on 1) comprehensively considering the various influencing factors (such as chemical stability, Li ion conductivity, mechanical strength, and flexibility) that affect the cycling performance of lithium metal anodes, the promising direction in the future is to design and construct organic/inorganic SEI layers with rational structure and accurate composition; 2) developing scalable methods with low cost to address the bottleneck of lithium metal anodes and realize their commercialization; 3) developing advanced characterization technology, especially *in situ* characterization technology, to monitor the SEI formation and evolution process; 4) comprehensively simulating an actual environment of LMBs in practical application, which could accurately evaluate the cycling stability under sudden environment changes, and the ability of resisting internal/external pressure and temperature changes under special conditions; and 5) developing effective simulation and calculation methods, accurately predicting the cycle life under the actual use conditions and designing reliable safety performance evaluation parameters, which can eliminate potential security risks in time.
